# Single-Pulse Response LA-ICP-MS Imaging with Quadrupole Instrumentation: Theoretical Considerations and Practical Assessment

**DOI:** 10.1021/acs.analchem.5c01870

**Published:** 2025-07-02

**Authors:** Jakob Willner, Lukas Brunnbauer, Maximilian Podsednik, David K. Gibbs, Ricarda Kriechbaum, Oliver Spadiut, Andreas Limbeck

**Affiliations:** † Institute of Chemical Technologies and Analytics, Research Group for Surface Analytics, Trace Analytics and Chemometry, 27259 TU Wien, Getreidemarkt 9/164-I2AC, Vienna 1060, Austria; ‡ Institute of Chemical, Environmental and Bioscience Engineering, Research Division Integrated Bioprocess Development, TU Wien, Gumpendorferstraße 1a, Vienna 1060, Austria

## Abstract

Recent instrumental developments in LA-ICP-MS, such as rapid response cells that significantly reduce the washout and the introduction of high-repetition rate lasers, have greatly impacted the field. To fully take advantage of these developments, improvements on the ICP-MS side were necessary. This was achieved by establishing ICP-TOF-MS instruments enabling simultaneous multielement detection suitable for recording the short transient signals generated by modern rapid response cells. These developments enabled a new operational mode for imaging called the single-pulse response (SPR). Nevertheless, quadrupole-based ICP-MS (ICP-Q-MS) instruments are still the most prevalent instrument type nowadays. Even though it provides many benefits, SPR-based imaging has not yet been applied to ICP-Q-MS due to the sequential *m*/*z* detection. However, recent developments enabling shorter dwell times and settling times of ICP-Q-MS systems make a SPR-based imaging approach feasible. In this work, we investigate the potential and limitations of SPR-based imaging using ICP-Q-MS. Therefore, we investigate the relationship between the cycle time and the signal stability by evaluating ^107^Ag/^109^Ag while ablating NIST SRM 612. With the results derived, theoretical considerations about the relationship between the peak width, laser repetition rate, and dwell times can be confirmed. In the next step, the effects of different dwell times on image quality and image artifacts are evaluated by imaging a test structure. Finally, the applicability of the SPR-based LA-ICP-Q-MS imaging approach is demonstrated for life science applications by showing SPR multielement measurements of cell samples, detecting 4 elements with a pixel acquisition rate of 100 Hz.

## Graphical abstract

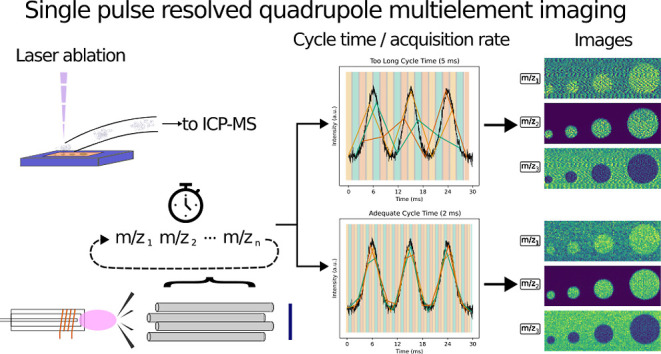

## 1 Introduction

Since the first reports of Laser Ablation-Inductively Coupled Plasma-Mass Spectrometry (LA-ICP-MS) in 1980,^1^ elemental mapping, often just called imaging, has become one of its major applications in fields such as bio/life science, geology, and materials science.^2,3^ Over the past few years, there has been a tremendous amount of instrument development impacting the field of LA-ICP-MS imaging. With advanced ablation cell designs (rapid response cells), washout times have been reduced to the low ms range, enhancing the sensitivity and reducing measurement times due to faster pixel acquisition rates.^4^ Further developments in laser technology increased available repetition rates up to the kHz range, which enabled measurement of high-resolution images in a reasonable time. To fully take advantage of these developments on the LA side, improvements on the ICP-MS side were necessary. This was achieved by the development and implementation of time-of-flight-based instruments (ICP-TOF-MS). These instruments enable true simultaneous multielement detection, making them an ideal complement to fast lasers and rapid response cells. Even though the combination of LA with an ICP-TOF-MS instrument provides many benefits for imaging, quadrupole-based instruments (ICP-Q-MS) are still by far the most widely used instrumental setup due to the lower cost and better sensitivity. Nevertheless, due to their sequential detection of predefined *m*/*z* values, ICP-Q-MS instruments cannot fully exploit the benefits facilitated by modern rapid response cells and high-repetition rate lasers.

Thus, in conventional LA-ICP-Q-MS imaging, the most commonly used approach is scanning parallel lines with overlapping laser shots, producing a plateau-like signal (continuous scan).^5–9^ Despite providing advantages such as multielement capabilities, this imaging approach inherently introduces a blur to the resulting image, which cannot be avoided due to the stage moving during the acquisition of an individual pixel. Additionally, typically large numbers of laser shots are fired, introducing significant amounts of material into the ICP-MS, which is not favorable in terms of instrument maintenance and component lifetime. Moreover, with this approach, a homogeneity of the sample into a certain depth, which increases substantially with a higher number of overlapping laser shots, is required or must be assumed.

Besides this conventional approach, in the field of LA-ICP-TOF-MS, a different imaging strategy has been developed based on single-pulse response (SPR).^10,11^ In this case, only one laser shot is fired per acquired pixel. This enables a clear correlation between the obtained signal and the spatial position of the analyzed sample, avoiding artifacts in the image. Furthermore, with this approach, the just mentioned constraints about the high depth homogeneity requirements are significantly reduced compared to the conventional procedure using multiple overlapping shots. Additionally, the application of modern rapid response cells and high-repetition-rate lasers is enabled, significantly increasing the pixel acquisition rate. Recently, a pixel acquisition rate of 1 kHz was demonstrated for the first time in SPR-based LA-ICP-TOF-MS imaging.^12^ Nevertheless, this novel approach requires dedicated software for data evaluation.

Even though SPR-based imaging provides many benefits, it has not yet been applied to LA-ICP-Q-MS instruments as a consequence of the slower sequential detection of isotopes and the limited time available to detect all elements of interest during the short transient signal derived from a single laser pulse in a rapid response cell. Nevertheless, recently introduced ICP-Q-MS instruments offer significantly reduced dwell times per isotope and reduced settling times of the quadrupole, allowing for faster switching and detection of multiple *m*/*z*, which opens the possibility to apply SPR-based analysis. The applicability of this approach has recently been demonstrated by Podsednik et al.^13^ reporting depth profile measurements for two elements. In this work, we investigate the potential but also limitations of a SPR-based imaging approach using a state-of-the-art ICP-Q-MS instrument in terms of multielement capabilities and speed of the analysis.

## 2 Experimental Section

### 2.1 Samples and Preparation

The glass reference material NIST SRM 612 (Gaithersburg, MD, USA) was used without any further preparation for the investigations on the influence of parameter variations on the precision of the measurement data in a single-pulse response acquisition. For the demonstration of a multielement imaging analysis, an artificial sample has been prepared using pulsed laser deposition and photolithography in combination with ion etching, as described by Schraknepper et al.^14^ The microstructured sample contained seven circles with different diameters ranging from 20 to 200 μm. The circles were deposited as an approximately 100 nm thin layer of SrRuTiO_3_ onto a SrTiO_3_ substrate.

 (UTEX 2505) used in this study was obtained from the Culture Collection of Algae at the University of Texas at Austin. was cultivated at consecutive 14 h light (100 μmol/m^2^/s) and 10 h darkness cycles at 20 °C in 3.0% (v/v) CO_2_-enriched air in a Minitron shaker (Infors HT, Basel, Switzerland). The utilized medium was BG11 at 1.0 g/L NaNO_3_.^15^ Thirty milliliters of BG11 medium were sterilely added to a 100 mL shake flask prior to inoculation with to a starting OD_600_ value of 0.1. The initiation of secondary carotenoid production was induced through nutrient limitation (nitrogen and phosphorus) after 3 weeks of cultivation, resulting in a nonmotile cell stage which was approximately 30–50 μm in size. These nonmotile cells were washed with an aqueous 0.9% (v/v) NaCl solution and diluted to OD_600_ values of 1.0 prior to heat fixation on glass slides (Carl Roth, Karlsruhe, Germany).

### 2.2 LA-ICP-MS Measurements

The measurements were performed with an “imageGEO193^LIBS^” laser ablation system from Elemental Scientific Lasers (Bozeman, MT, USA) operating at a wavelength of 193 nm and equipped with a “TwoVol3” ablation chamber. The instrument was coupled to a “NexION 5000” Multi-Quadrupole ICP-MS system from PerkinElmer (Waltham, MA, USA), using PEEK tubing and a “Dual Concentric Injector” (DCI) interface (ESL, Bozeman, MT, USA). The instrument parameters of the laser ablation system and ICP-MS were optimized to enable washout times in the low millisecond range. All experiments were performed using line scans as ablation patterns with nonoverlapping shots, a chamber gas flow of 200 mL/min He, and a sniffer gas flow of 250 mL/min He. The ICP-MS was operated using a plasma power of 1600 W and a nebulizer gas flow of 1200 mL/min Ar. Additional instrumental parameter ranges are shown in the Supporting Information, Table S1, and the exact parameters for each experiment are given in the respective results sections and in the Supporting Information, Table S2. Before all experiments, the ICP-MS instrument was tuned for a maximum ^115^In signal while ablating a NIST SRM 612. ICP-MS data was recorded using Syngistix 3.5 provided by the manufacturer.

### 2.3 Data Evaluation

Due to the novelty of the investigated SPR-based LA-ICP-Q-MS imaging approach, no commercial software solution is available for data evaluation. Therefore, data evaluation was carried out with a custom Python-based Jupyter Notebook aiming to compensate for the lack of synchronization information on the laser pulses with the MS signal. The data evaluation aims to integrate the signals resulting from each laser shot. When running imaging experiments, the number of expected peaks in the transient signal corresponds to the number of pixels in the image, which is equal to the number of laser shots fired. It is essential that the correct number of peaks, as well as their correct timestamp, is identified in the transient signal; otherwise, pixels will be missing and the image cannot be correctly reconstructed based on the data. In some cases, a laser shot may not produce a signal for any of the elements detected (e.g., due to the elements not being present at that particular position). Therefore, simply using a peak-finding algorithm to identify all of the peaks in the transient signal is not a feasible approach.

The applied approach to reliably identify the correct positions of peaks in the transient signal is based on autocorrelation with a template signal for each line scan. In the first step, an idealized template signal is generated based on the number of laser shots in a single line scan using a sawtooth signal with the same sampling rate as the ICP-MS signal. In the next step, an autocorrelation between the template signal and the ICP-MS signal is calculated. We now expect a number of maxima in the correlation to be equal to the number of line scans measured. These are the positions where the template signal correlates the highest with the transient signal for each line scan (Supporting Information Figure S1). The position of the peak maxima in the correlation is identified using scipy.signal.find_peaks.

We can now use the positions of the template signal to set the integration limits for each peak in the transient signal (Figure 1). With this approach, we can ensure that the right number of peaks at the correct position in the transient signal is evaluated.

**Figure 1:**
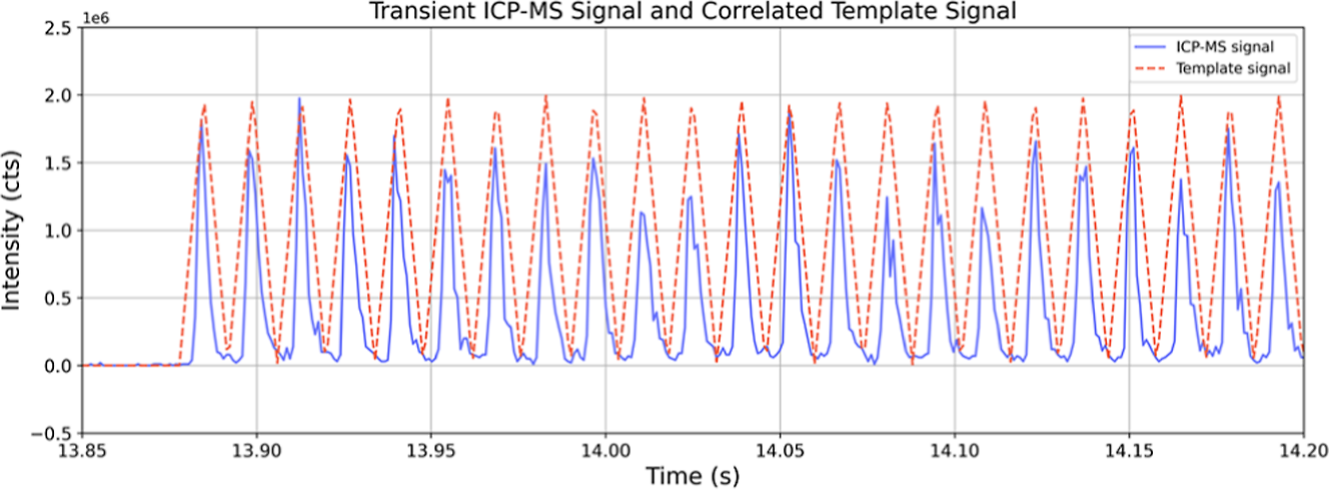
Overlap of the template signal with the transient ICP-MS signal showing that the peak positions are aligned.

## 3 Results and Discussion

### 3.1 Theoretical Aspects for Recording Short Transient Signals with ICP-Q-MS

As described in the introduction, ICP-Q-MS instruments sequentially record data for different *m*/*z*. This poses a problem when the signals to be recorded are short transient signals. The important metrics when recording those signals are as follows:

- Settling time: the time it takes for the quadrupole to stabilize when switching between *m*/*z*
- Dwell time: the time the quadrupole is set to a specific *m*/*z*, recording the signal of the selected *m*/*z*
- Cycle time: the time it takes to cycle through all selected *m*/*z* including settling and dwell times
- Duty cycle: Percentage of the sum of all dwell times to the total cycle time^16^

The peak width of a typical transient signal produced by modern LA systems used for SPR imaging is in the range 1–20 ms. Therefore, to enable multielement analysis with a quadrupole-based system, the settling time and the dwell times of the individual *m*/*z* must be selected accordingly short to get a cycle time that enables a sufficient signal sampling rate with a precision fit for reconstructing the peaks for all elements. Figure 2 shows scenarios for two different cycle time durations for simulated SPR signals with a base-to-base peak width of 10 ms. The black signal corresponds to the “true” signal, whereas the red, green, and yellow signals correspond to the acquired signals of 3 different *m*/*z*. Recording the data with an adequate cycle time (Figure 2a) results in 5 data points per *m*/*z* per peak. Recording the same data with a too long cycle time (Figure 2b) results in a reduced number of data points per peak, which may inflict image artifacts such as aliasing. It should be noted that simply using shorter dwell times is not sufficient since, in this case, the amount of actual measurement time per cycle time (i.e., duty cycle) decreases, thereby hampering counting statistics and sensitivity. Hence, the application of short settling times is crucial. Fast ICP-Q-MS instruments provide settling and dwell times in the range of several hundred μs enabling short cycle times. With this, an adequate measurement of short transient signals is feasible while detecting multiple *m*/*z*.

**Figure 2:**
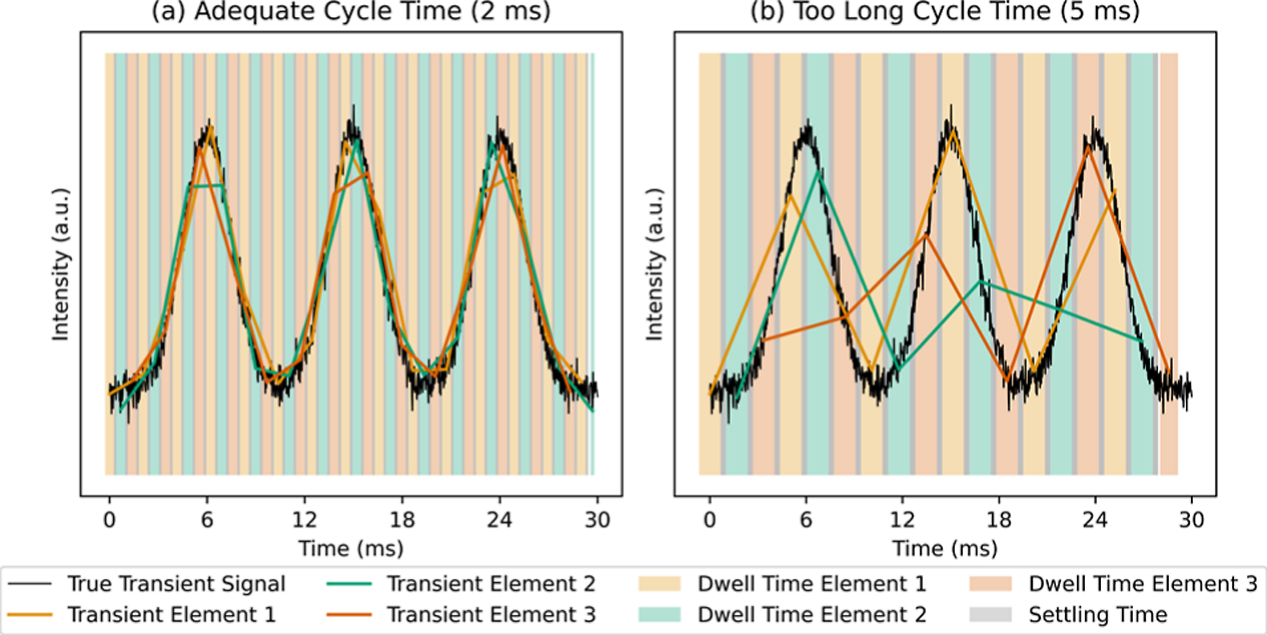
Simulated SPR transient signals showing the influence of the ICP-MS cycle time on data acquisition. (a) Using an adequate cycle time resulting in 5 data points per *m*/*z* per peak and (b) using a too long cycle time resulting in inadequate sampling.

In this work, we consider 5 data points per peak sufficient to calculate peak integrals as this results only in an error of 8.5% considering ideal Gaussian shaped peaks (see Supporting Information—Figure S2). If higher precision is necessary, then the number of data points per peak must be increased. Therefore, theoretical considerations about the number of elements detectable with certain dwell times, settling time, and specific peak widths (and thus repetition rate and pixel acquisition rate) are possible (Figure 3). Assuming a fixed settling time of 0.2 ms, a dwell time of 0.2 ms per *m*/*z*, and a peak width of 8 ms, theoretically 4 elements can be detected with a sampling rate of 5 data points per peak, resulting in a maximal repetition rate of 125 Hz.

**Figure 3:**
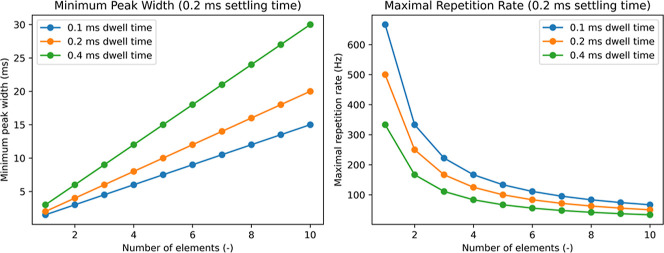
Theoretical considerations regarding the number of elements that can be detected in relation to the minimum peak width (ms) left and the resulting maximal repetition rate (Hz) right for different dwell times per element.

Moreover, in addition to the discussed theoretical considerations, some practical requirements must be considered. It should be mentioned that the dwell time has a direct impact on the signal-to-noise ratio and it may have to be selected in such a way that sufficient S/N is obtained. Further, the fraction of the dwell time of a specific isotope to the cycle time is proportional to the number of ions counted during a transient signal, thus influencing the sensitivity. Additionally, the sensitivity depends on the spot size, which influences the peak width. Tuning and adjusting the peak width accordingly is crucial as it is a prerequisite for multielement analysis. Narrow peaks typically improve sensitivity, but, for multielement analysis with quadrupole ICP-MS, broader peaks are necessary to record sufficient data points per element. In summary, all of the parameters (peak width and shape, settling time, dwell times, number of detected elements, spot size, laser energy, and required sensitivity) influence each other and must be carefully optimized. Adjusting the experimental settings for increased sensitivity may lead to longer measurement times compared to the best-case theoretical scenario. In addition, differences in peak shapes (width and skewness) among major, minor, and trace constituents must be accounted for when determining the optimized experimental settings.

Therefore, SPR-based multielement imaging with quadrupole ICP-MS is possible if the theoretical requirements discussed in this chapter are considered and the practical requirements are thoroughly optimized and adjusted to the specific tasks’ needs. However, theoretical considerations in this section assume ideal peak shapes. Subsequently, the error derived from interpolation between individual data points would be minimized. In reality, peaks are typically not center-symmetrical, non-Gaussian, etc.,^17^ and peak width may vary between different sample locations due to sample properties or changes in the washout caused by changes in the gas fluid dynamics derived from sample chamber location or sample surface morphology. Additionally, software capable of evaluating SPR data recorded with an ICP-Q-MS instrument is, to the best of our knowledge, not readily available. Even though modern LA systems provide a trigger signal for each laser shot, facilitating correlation of the obtained signal with the corresponding laser shot, this feature is only used in ICP-TOF-MS systems. If a synchronization between the laser and the ICP-MS instrument is not available, more complicated data evaluation strategies enabling the extraction of the corresponding ICP-MS signal for each individual laser shot must be considered.

### 3.2 Experimental Determination of the Acquisition Parameters’ Influence on the Precision Using NIST SRM 612 (Shot-to-Shot Variations)

Despite the theoretical considerations shown in the previous chapters already giving a good estimate regarding the acquisition parameters’ requirements for performing SPR multielement analysis with sequentially measuring quadrupole instruments, experimental data is indispensable to allow for a quantitative assessment of the resulting error margins. To demonstrate this, NIST SRM 612 was used to measure Ag, which is present at a concentration of 22.0 ± 0.3 μg/g,^18^ under varying cycle times. This is achieved by varying the dwell times and the total number of detected isotopes (influencing the ratio of each individual dwell time per cycle time). With each measurement parameter setting, 150 pulses (3 J/cm^2^ fluence, 20 × 20 mm^2^ spot size, 100 Hz repetition rate) were recorded and evaluated, which corresponds to approximately 2 fg of silver per pulse based on the ablated mass and Ag concentration in the NIST glass.

The applied instrument’s “nano module” allows the acquisition of a single isotope at ultrashort dwell times (between 10 μs and 50 ms), without any switching between elements and thus without any settling times between the data points. As a result, the “true” peak shape, width, and integrated intensity can be assessed without interpolation or corrections being performed. The results are shown and discussed in the Supporting Information (Figure S3), with a strong focus on the peak-to-peak intensities and the RSDs between the integrated peaks using different measurement dwell times. For the obtained peak widths of 7–8 ms, the RSD of the integrated peaks of 5.4% with the best conditions can be considered as a reference for the other measurements that contain more than one isotope. Since the “nano module” offers 100% signal acquisition for one isotope (no settling time), the RSDs observed there should directly reflect the sample homogeneity and variations of the surface morphology, laser stability, and variations in transport efficiency.

All further measurements, including more than one isotope, were performed in standard measurement mode. Thus, each acquired data point is preceded by a quadrupole settling time of 200 μs. After starting with the measurement only of ^107^Ag and increasing the dwell time by 100 μs increments, ^109^Ag was added as a second isotope and dwell times were increased stepwise from 100 to 600 μs. This was repeated after adding ^88^Sr and finally, also ^138^Ba. Since different dwell times and numbers of measured isotopes result in a varying amount of data points per peak and different ion counts per data point, the integrated peaks must be corrected according to the dwell time per cycle time to enable a comparison of the average peak integrals between different measurement conditions. This was done for the data shown in Figure 4. Nevertheless, the key factor is the relative standard deviation within 150 measured peaks in each measurement condition, rather than comparison of the absolute average peak integral between different measurement conditions, since the RSD would reflect the measurement parameters’ influence on the precision, which directly reflects the pixel-to-pixel variation of an imaging experiment.

**Figure 4:**
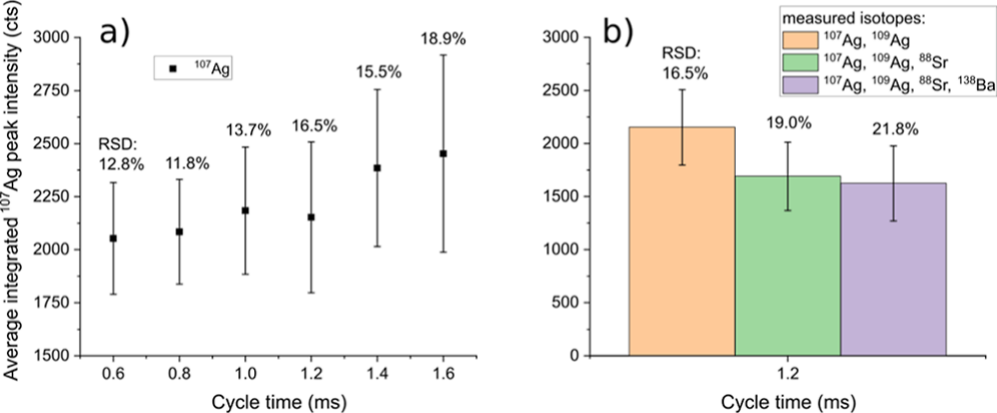
^107^Ag average integrated peak intensity from NIST SRM 612 measurements at different dwell times, cycle times, and number of measured elements. (a) Measurement of ^107^Ag and ^109^Ag with increasing dwell times. (b) Measurement of varying numbers of isotopes (2–4) at constant cycle time.

Figure 4a shows the average integrated ^107^Ag intensity when ^107^Ag and ^109^Ag are measured at dwell times from 0.1 to 0.6 ms and a constant settling time of 200 μs resulting in cycle times from 0.6 to 1.6 ms. The RSDs within one condition vary between 11.8% at a 0.6 ms cycle time and 18.9% at a 1.6 ms cycle time, which is higher than obtained with the nanomodule (5.4–9.6%, with 75 μs dwell time and 25 μs dwell time, respectively, discussion seen in the Supporting Information). This is a result of the actual ^107^Ag acquisition of each data point (i.e., individual dwell time) being only a fraction of the total measurement time (i.e., cycle time), thus leading to a decreasing duty cycle and increasing imprecision when acquiring transient signals. Furthermore, the trend shows increasing RSDs with increasing cycle times, which is explained by the decreasing sampling rate and the resulting number of data points per peak. An exception of this trend is observed for an RSD of 11.8% at 0.8 ms being slightly lower than that at 0.6 ms, which could be explained by the lower counting statistics and lower actual measurement time per cycle time at the lowest dwell time. The counting statistics influence on the results is further discussed in the nanomodule’s measurements in the Supporting Information.

Figure 4b shows the average integrated ^107^Ag intensity at a constant cycle time of 1.2 ms when two, three, or four isotopes are measured. Including 200 μs of settling time per isotope, this corresponds to dwell times of 0.4 ms for two measured isotopes, 0.2 ms for three isotopes, and 0.1 ms for four isotopes. While the sampling rate and thus average number of data points per peak are identical, the individual dwell time per cycle time for each measured *m*/*z* decreases when an increasing number of isotopes are measured, leading to the increase of the RSDs from 16.5% to 21.8%.

In summary, the results from Figure 4 show that the peak-to-peak RSD increases with increasing cycle time (i.e., decreasing number of data points per peak) and with decreasing measurement time of the isotopes (i.e., duty cycle). For the presented case showing 7–8 ms peak widths, using single isotopic acquisition with the “nano module” peak-to-peak RSD can be as low as 5.4%. This case is not influenced by the working principle of quadrupole instruments (sequential measurement of isotopes preceded by settling times) and thus directly resembles the homogeneity of the sample and the stability of the instrumentation (sample surface variations and homogeneity, laser energy stability, aerosol washout and transport, stability of the plasma, ion transmission, etc.). With two isotopes, observed RSDs are above 10%, and with three or four isotopes in the range of 15–20%. Since in an imaging experiment, each peak would resemble a pixel, the observed RSDs would resemble the noise of an ideally constantly intense feature in the image (assuming the NIST SRM 612 being perfectly homogeneous on a scale of the used spot size of 20 μm and neglecting variations in the laser output fluence and differences in ablation due to surface variations of the sample). It is shown that the peak-to-peak RSDs can be as low as approximately 12% at measurements of multiple isotopes in standard measurement mode.

Figure 5 shows data from the same measurements, as shown in Figure 4. However, instead of evaluating the ^107^Ag integrated peak intensity as previously discussed, the ^107^Ag fraction calculated as ^107^Ag/(^107^Ag + ^109^Ag) is shown, thus including normalization, which is commonly applied in LA-ICP-MS whenever possible. The natural abundance value of the ^107^Ag is indicated with the dashed grey line. It can be seen that the RSDs in Figure 5a follow the same trend as in Figure 4a, increasing with higher cycle times, with the exception being observed for the lowest dwell time. However, overall, the RSDs ranging from 5.1 to 13.9% for the normalized data are substantially smaller compared to the non-normalized ^107^Ag integrated peak intensities. Nevertheless, the influences of the sampling rate and the duty cycle are identical in this case.

**Figure 5:**
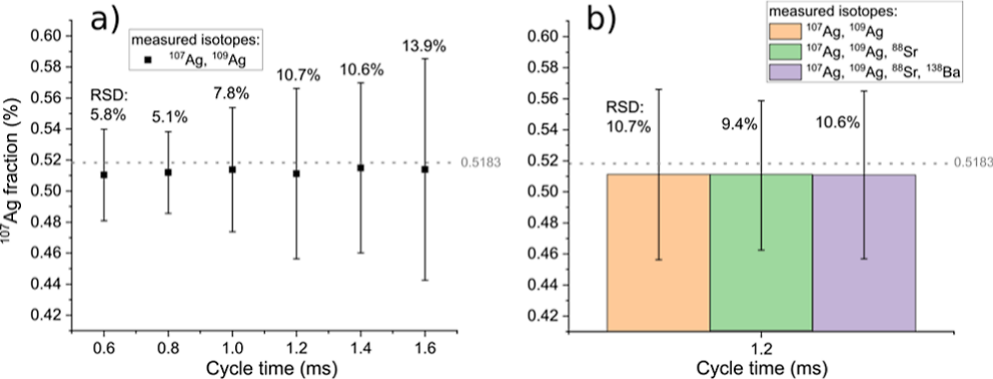
^107^Ag integrated intensity fractions and RSDs from NIST SRM 612 measurements at different dwell times, cycle times, and number of measured elements. (a) Measurement of ^107^Ag and ^109^Ag in standard mode with increasing dwell times. (b) Measurement of varying numbers of isotopes (2–4) in standard mode at constant cycle time.

Moreover, the RSDs of the average ^107^Ag fraction measured at a constant cycle time of 1.2 ms when a different number of isotopes are measured with different dwell times are nearly identical, ranging from 9.4 to 10.7%. This demonstrates that the variations observed for ^107^Ag integrated intensity RSDs in Figure 4 are compensated through normalization when the ^107^Ag fraction was calculated, as shown in Figure 5.

Concluding the investigation of the influence of varying acquisition parameters on single-pulse response measurements and evaluation demonstrated using NIST SRM 612, the errors derived from different influences were shown quantitatively and compared with each other. It was shown that normalization significantly improves the error margins and thus should be applied whenever possible. It is revealed that a careful optimization of the acquisition can keep the pixel-to-pixel RSD below 10%, which is an absolutely satisfying result for LA-ICP-MS in general and for high-resolution imaging applications of trace constituents especially. Finally, it must be mentioned that the presented data would correspond to a pixel acquisition rate of 100 Hz, which exceeds state-of-the-art published literature for quadrupole LA-ICP-MS imaging by a factor of 5 to 10.

### 3.3 SPR Multielement Imaging of a Sample with a Defined Microstructure

To perform a practical investigation of the acquisition parameters’ influence on the resulting images, a technological microstructured SrTiO_3_/SrRuO_3_ thin film sample was analyzed. The material combination used as the test structure is of high interest in electrochemical applications, where it finds use in a variety of all-oxide electronic devices due to its high electronic conductivity, and small lattice mismatch with other perovskite oxides, making it an ideal electrode material.^14^ One of the most prominent applications is as cathode material in solid oxide fuel cells (SOFC), where they provide efficient oxygen reduction and high-temperature stability and reliability.^19^

In accordance with the theoretical considerations and the insights gained from the NIST measurements of the previous section, the laser ablation parameters were optimized to receive satisfying peak widths while maximizing spatial resolution (i.e., spot size), thus enabling a successful imaging of three elements (Sr, Ru, and Ti) at the highest possible pixel acquisition rate. Preliminary experiments and optimization resulted in peak widths of approximately 10–15 ms which should lead to satisfying results for cycle times below 2 ms. Considering the tolerance margins mentioned above, a laser repetition rate of 70 Hz was chosen to record the images, which ensured baseline separated peaks. A spot size of 2 × 2 μm^2^ and a fluence of 1.2 J/cm^2^ were used together with the standard instrument settling time of 200 μs and dwell times from 0.1 to 0.6 ms for the measurement of ^84^Sr, ^49^Ti, and ^96^Ru. Using a low laser fluence is particularly important in this case to allow proper differentiation between the thin films and the substrate since the thickness of the deposited SrRuO_3_ circles is only around 100 nm. Figure 6 shows a microscopic image (a) and schematic side view (b) of the sample, which was prepared as described in Section 3.1. The signal (cycle time of 1.2 ms) of a small section measured over the interface, as marked with a red line in Figure 6a, is shown in Figure 6c.

**Figure 6:**
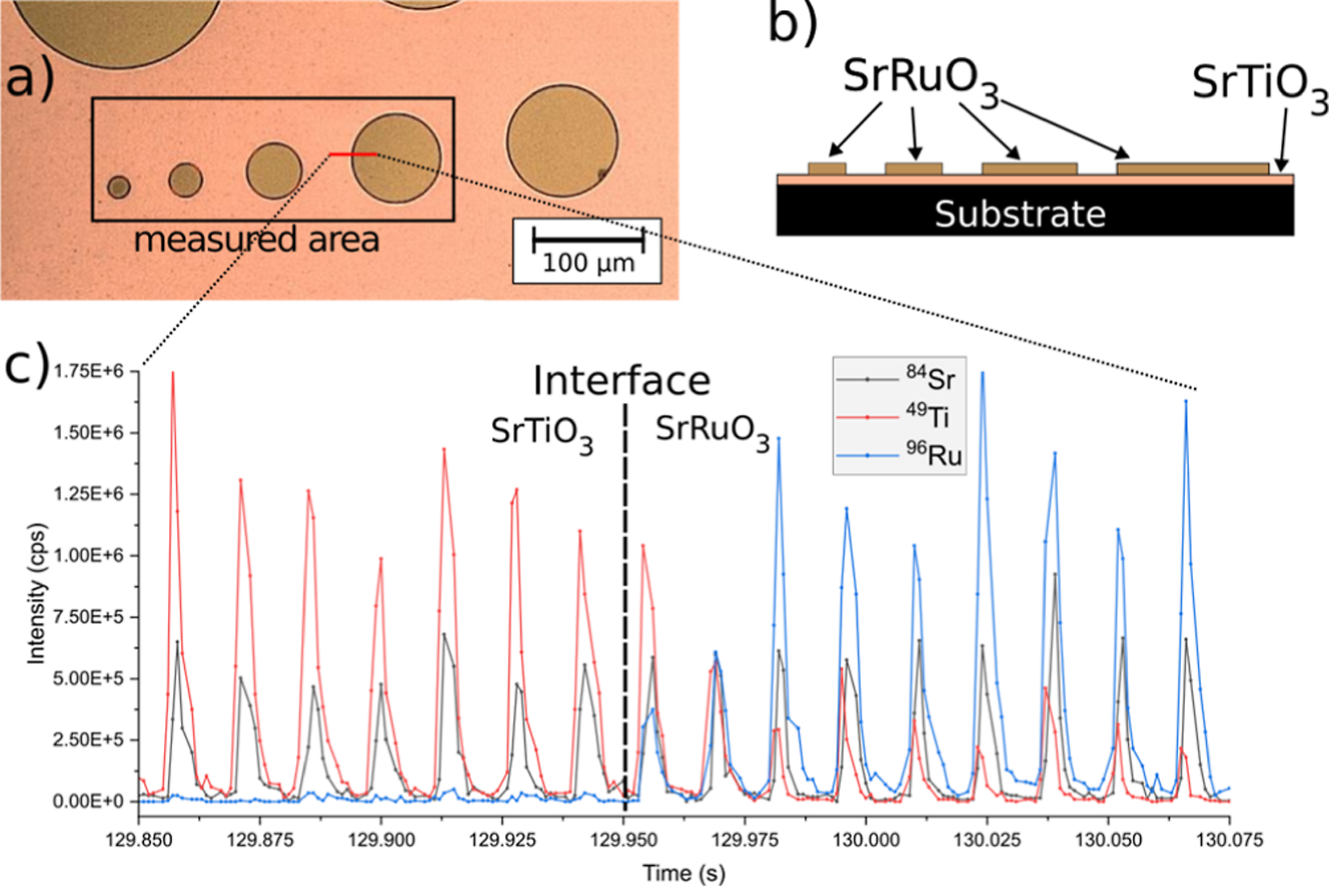
Depictions of the SrTiO_3_/SrRuO_3_ test structure used for SPR multielement images: (a) microscopic image in 200× magnification, (b) schematic drawing—side/top view, and (c) measured signal over the SrTiO_3_/SrRuO_3_ interface.

Elemental mappings of the area marked in Figure 6a were measured by using the above-mentioned ablation parameters. Figure 7a shows the elemental mappings of two consecutively ablated layers (on the same sample position) for Sr, Ru, and Ti, measured from the structured SrRuO_3_ circles on the SrTiO_3_ structure using dwell times of 0.1 ms for each isotope, which results in a cycle time of 0.9 ms. The elemental mappings are in good accordance with the sample structures (Figure 6). It is observed that in reference to the image contrast, Sr originating from the SrRuO_3_ can be distinguished from that of the SrTiO_3_ phase, though it is present in both phases in the same amount from a stoichiometric perspective. This shows the influence of the two different materials on the ablation and the resulting signal intensities, demonstrating matrix effects on a high spatially resolved level (2 μm laterally, 100 nm thin layer). As expected, Ru is seen only on the circular film deposited on top of the titanate film (the schematic sample structure is shown in Figure 6b). Finally, the Ti images show the negative image compared to the Ru images, i.e., higher intensities outside of the circles where ruthenate was deposited. Furthermore, it is seen that the SrRuO_3_ layer was nearly completely ablated in the first layer, which demonstrates the advantage of the SPR imaging approach without overlapping laser shots for thin film structures. In the second ablated layer, Sr is barely visible because of the lower contrast, Ru is still slightly visible because of the high contrast and signal-to-background ratio, and for Ti, no contrast can be seen in the image (Figure 7a).

**Figure 7:**
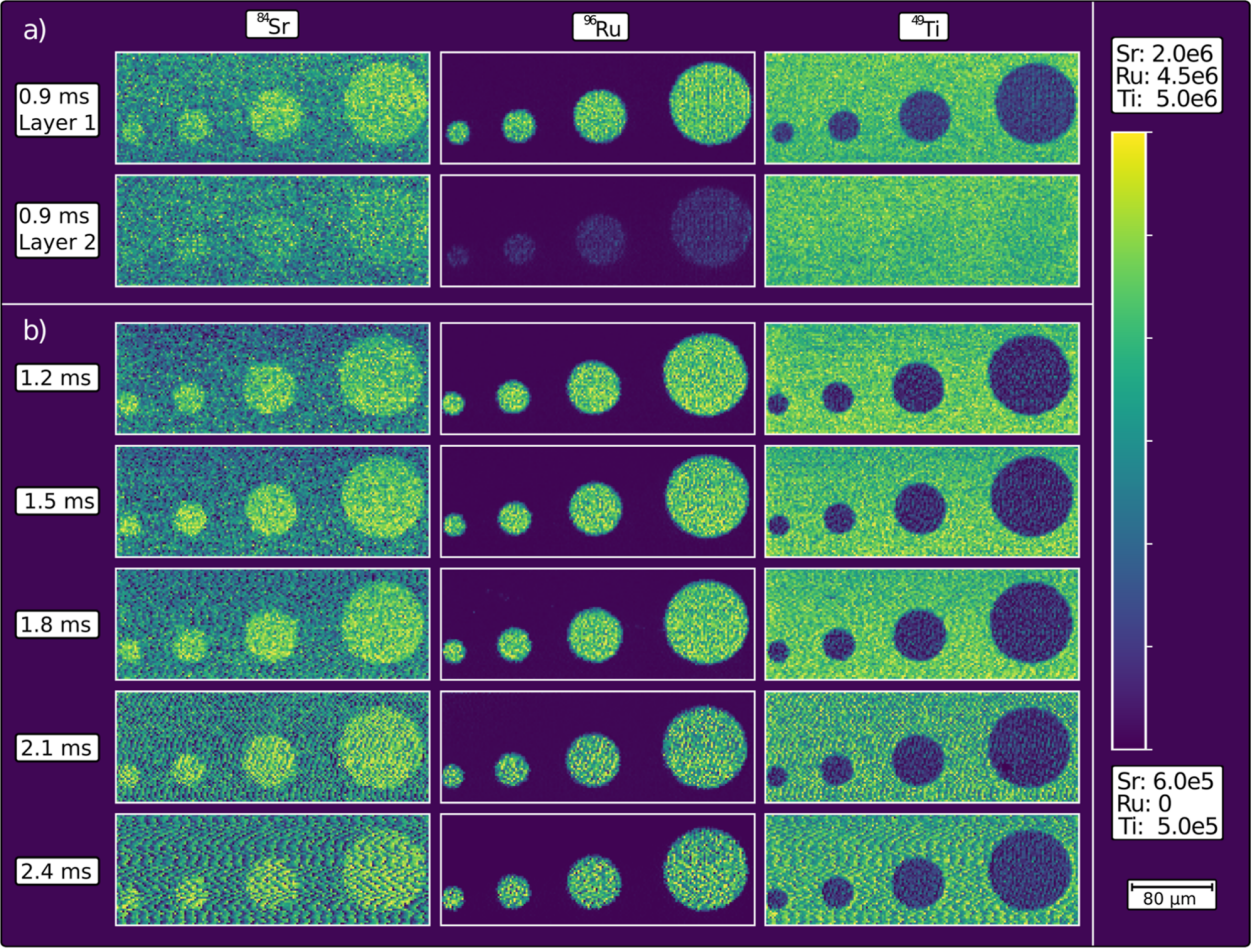
Elemental mappings for Sr, Ru, and Ti of the test structure measured using different dwell times and resulting cycle times with individual color scale ranges for each element. (a) shows the first and the second ablated layer measured with a 0.9 ms cycle time and (b) shows the first ablated layer with increasing cycle times.

However, another aspect crucial for image quality has not yet been discussed. Since the laser pulses are not synchronized with the cycle time of the ICP-MS, aliasing can occur if the data acquisition rate is not high enough to fully represent the transient peaks, which is expressed in the resulting images through patterns of oscillating intensity in areas where a constant signal with constant noise would be expected. This phenomenon, well-known from signal theory, is described by the Nyquist theorem, according to which the acquisition rate must be at least double the fastest occurring signal frequency. However, this cannot directly be transferred to the presented case since it refers to sine waves rather than the peak shapes observed in our case. Thus, if the conditions (peak widths versus cycle time) are close to theoretical limitations, then experimental confirmation is required to ensure proper imaging without aliasing.

Figure 7b shows the first ablated layer measured with increasing dwell times (0.2–0.6 ms) and, thus, cycle times (1.2–2.4 ms). The geometry of the structure can be well-recognized with all measurement conditions due to the high image contrast. With an increasing cycle time, no significant differences are observed up to a time of 1.8 ms, where aliasing effects start to be visible in the background and significantly increase with higher cycle times. Within the circles, aliasing begins to be clearly visible only starting from a cycle time of 2.1 ms, which might be explained by the wavy aliasing patterns being more easily recognized by human perception on larger areas.

To enable a discussion of the pixel-to-pixel variation of the measured images, in each image, regions of interest (ROI) were defined as a circle of 80 μm in diameter. Figure 8 shows the RSDs of the ROIs of SrRuO_3_ (Figure 8a) and the SrTiO_3_ phase (Figure 8b). It is seen that using sufficiently short cycle times (<1.8 ms), the observed pixel-to-pixel RSDs within the ROIs are approximately 20% and lower. This lies within an expected range compared to the NIST 612 measurements, especially when considering that the image evaluation presented here was measured with a 2 × 2 μm^2^ spot size (corresponding to an ablated mass between 500 fg and 1 pg per laser pulse depending on the element and matrix) and without a suitable element for normalization being present in the sample. However, when the cycle time is increased above the limit, the RSDs increase significantly. Hence, it is demonstrated that the acquisition parameters must be carefully optimized.

**Figure 8:**
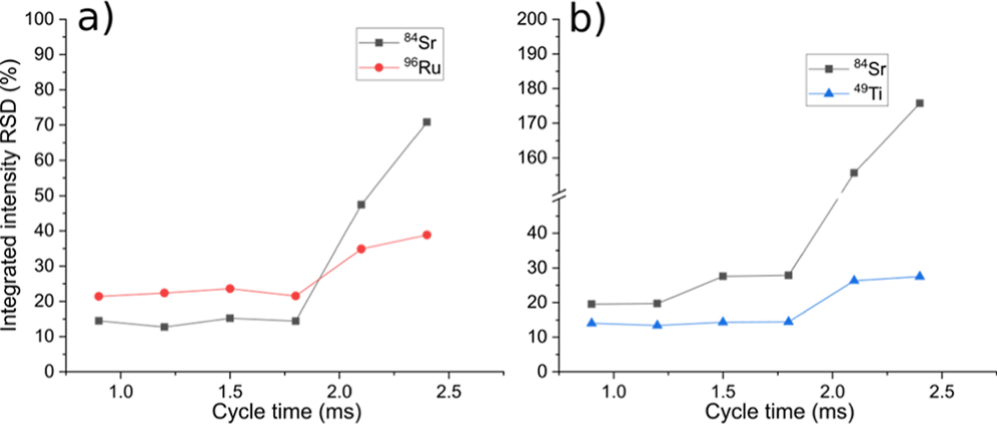
RSDs of the ROIs in the SrRuO_3_ phase (a) and the SrTiO_3_ phase (b) from the images measured with different cycle times.

### 3.4 SPR Multielement Imaging of a Biological Sample

To demonstrate the applicability of the proposed approach on biological materials, multielement imaging experiments were performed on () cell samples, cultivated in a shake flask, and deposited on glass slides via heat fixation. is the main host for biotechnological production of astaxanthin, a carotenoid belonging to the class of xanthophylls.^20^ Astaxanthin is commercially used as a cosmetic ingredient, dietary supplement, or in feed applications,^21^ as it displays antioxidative, anti-inflammatory, and antiapoptotic properties.^22^ Astaxanthin production from is stimulated by environmental stress (nutrient limitation, osmotic stress, photooxidative stress), which induces the transformation into an encapsulation stadium of the cells. Environmental stressors, like high light conditions, could potentially lead to cell death in microalgae followed by cells bursting. This then leads to the release of a considerable amount of debris into the cultivation medium.^23^ Elemental imaging of samples from such cell cultures helps to gain insights and improve our understanding of the influence of cultivation parameters on the cells.

These imaging experiments were conducted using a laser fluence of 2 J/cm^2^ to ensure complete ablation of the biological material, and acquiring ^31^P, ^63^Cu, ^26^Mg, and ^55^Mn, all using dwell times of 0.1 ms. The applied 5 × 5 μm^2^ spot size resulted in peak widths below 10 ms, thus enabling a measurement with a 100 Hz pulse frequency. To exclude signal contributions from the glass slide, the applied ablation parameters chosen showed no visual ablation from the glass slide.

A microscopic image and the elemental mappings for P, Mg, Mn, and Cu are shown in Figure 9. In the left half, three separate intact cells can be seen, in the middle, an agglomerate of three cells is present, and in the upper right, there is an agglomerate of two cells. Additionally, between the cells, a significant amount of small fragments and intracellular debris is deposited, which is visible in the microscopic image as well as in the elemental mappings. It can be observed that phosphorus and magnesium show higher intensities at the center of the cells and the medium between the cells, whereas a decreased intensity is observed at the cell walls. In contrast, copper shows slightly increased signals in the complete cell areas. The same is observed for manganese; however, for manganese, a spot with decreased intensity is observable at the center of each cell.

**Figure 9:**
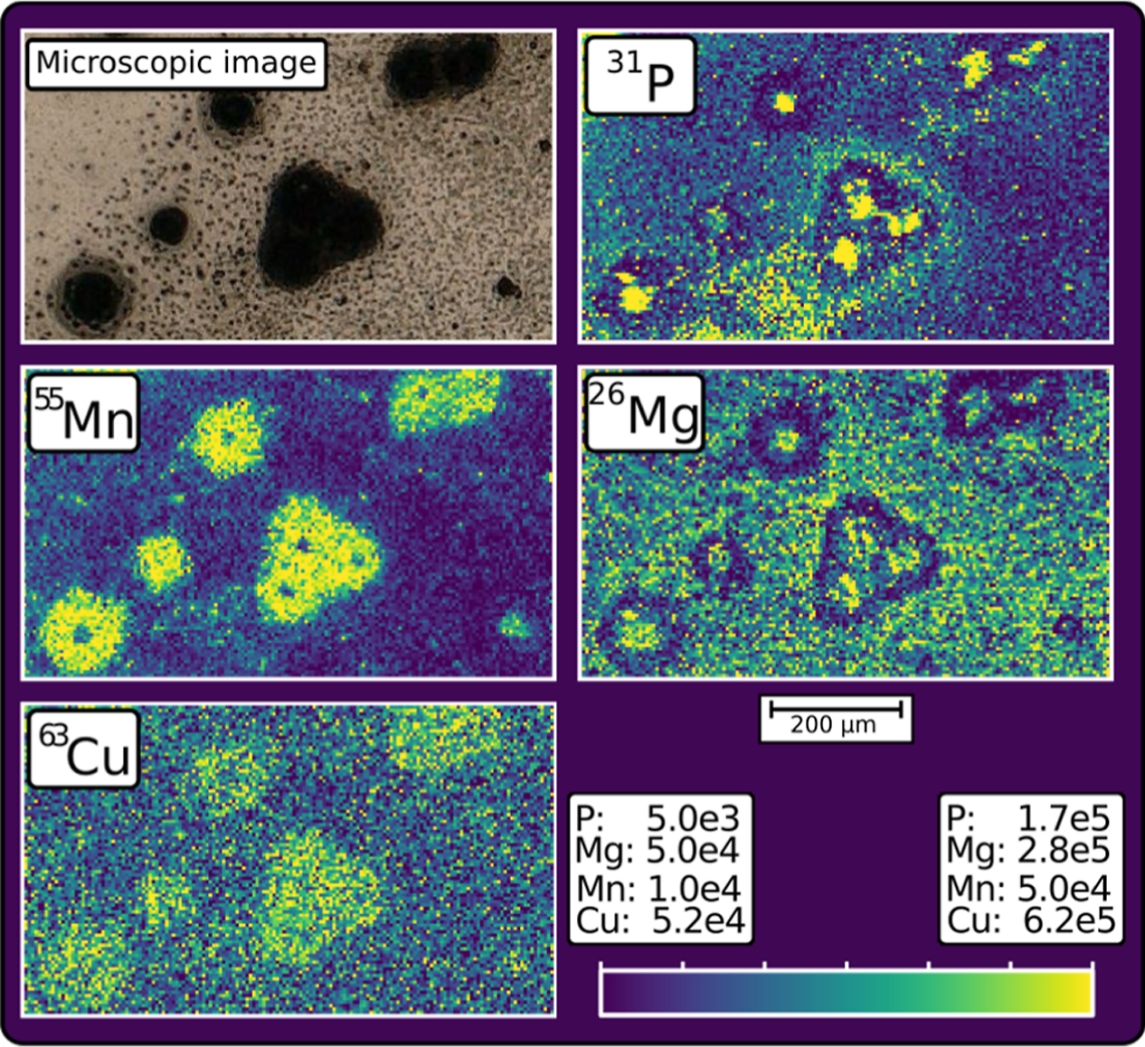
Microscopic image and elemental mappings for ^31^P, ^26^Mg, ^63^Cu, and ^55^Mn with individual color scale ranges for each element.

The presented images, which contain 158 × 92 pixels and were recorded in a measurement time of only four minutes, demonstrate the capability of the SPR approach with fast-switching quadrupole ICP-MS for imaging with high pixel acquisition rates in life science applications.

## 5 Conclusions

In this work, we investigate the application of modern ICP-Q-MS instruments for SPR-based LA-ICP-MS imaging. SPR-based imaging is gaining more and more attention due to the recent introduction of rapid response cells and ICP-TOF-MS instruments. While providing several advantages such as high surface sensitivity, no pixel bleeding, and significantly reduced measurement times, this approach was accessible only to ICP-TOF-MS instruments due to the high acquisition speed and simultaneous recording of full mass spectra. The recent introduction of fast quadrupole-based ICP-MS instruments opened the world of SPR-based imaging to ICP-Q-MS users. After theoretical considerations about instrumental requirements such as cycle time and peak widths of the SPRs, we evaluated these considerations by SPR measurements on NIST SRM 612 to quantify expected shot-to-shot variations for different dwell times and different numbers of *m*/*z* recorded. It was shown that using the best conditions (single isotope acquisition, no settling time), ∼5% RSD can be achieved. Further adding elements in standard measurement mode increases observed RSDs to approximately 10% when normalization is performed. Additionally, we imaged a defined test structure to verify these findings and visualized the effects of different cycle times. This already revealed that by using suitable parameters, 320 × 110 μm^2^ large images of three elements can be measured with a 2 × 2 μm^2^ pixel resolution and 70 Hz pixel acquisition rate in a measurement time below 3 min. Finally, we used SPR-based LA-ICP-Q-MS imaging for the first time for a life science application, demonstrating a pixel acquisition rate of 100 Hz while recording four elements. For imaging applications where only a selected number of elements are necessary, which is the case for a considerable amount of practical applications, especially in technological materials, SPR-based ICP-Q-MS imaging can provide superior sensitivity, boost the achievable spatial resolution, and reduce measurement time. However, for nontargeted analysis and research questions which require the analysis of 5 or more elements, still ICP-TOF-MS is the method of choice.

## Supplementary Material



## Notes

Additional experimental conditions, figure of the correlation plot, error in the Gaussian area depending on the number of sampling data points, and figure showing results from single isotope measurements with a discussion paragraph (PDF)
