# Supplementary material for: Single-Pulse Response LA-ICP-MS Imaging with Quadrupole Instrumentation: Theoretical Considerations and Practical Assessment
Source: Anal Chem. 2025 Jul 2;97(27):14521–30. doi: 10.1021/acs.analchem.5c01870 (PMC12268819; doi:10.1021/acs.analchem.5c01870)
Supplement: Supplementary file 1 [file ac5c01870_si_001.pdf]

Supporting Information

Single pulse response LA-ICP-MS imaging with quadrupole instrumentation: theoretical considerations and practical assessment

Jakob Willner[a], Lukas Brunnbauer[a], Maximilian Podsednik[a], David K. Gibbs[a], Ricarda Kriechbaum[b], Oliver Spadiut[b], and Andreas Limbeck[*a]

[a]TU Wien, Institute of Chemical Technologies and Analytics, Research Group for Surface Analytics, Trace Analytics and Chemometry, Getreidemarkt 9/164-I²AC, 1060 Vienna, Austria

[b]TU Wien, Institute of Chemical, Environmental and Bioscience Engineering, Research Division Integrated Bioprocess Development, Gumpendorferstraße 1a, 1060 Vienna, Austria

[*]Corresponding author email: andreas.limbeck@tuwien.ac.at

Table of Contents

Table S1. Overview of relevant experimental parameter ranges of the laser ablation and ICP-MS instruments.

Table S2. Relevant instrumental parameters and experiment characteristics.

Figure S1. Correlation of the template signal with the transient ICP-MS signal.

Figure S2. Relationship between the error of the integrated peak area (using the trapezoidal method) of a gaussian shaped signal depending and the number of recorded data points.

Figure S3. Average integrated 107Ag peak intensity and RSDs measured at different dwell times using the "nano module" (single isotope acquisition without settling times) on NIST SRM 612.

NIST SRM 612 measurements with „nano module".

Table S1. Overview of relevant experimental parameter ranges of the laser ablation and ICP-MS instruments.

**imageGEO193 Laser Ablation**	
Spot size	2 x 2 - 20 x 20 µm^2
Sample fluence	1.2 – 3.0 J/cm^2
Repetition rate	70 – 100 Hz
**NexION 5000 ICP-MS**	
Dwell times	100 – 600 µs
Settling time	200 µs
Measured Isotopes	^{107}Ag, ^{109}Ag, ^{88}Sr, ^{138}Ba, ^{49}Ti, ^{84}Sr, ^{96}Ru, ^{31}P, ^{63}Cu, ^{64}Zn, ^{55}Mn

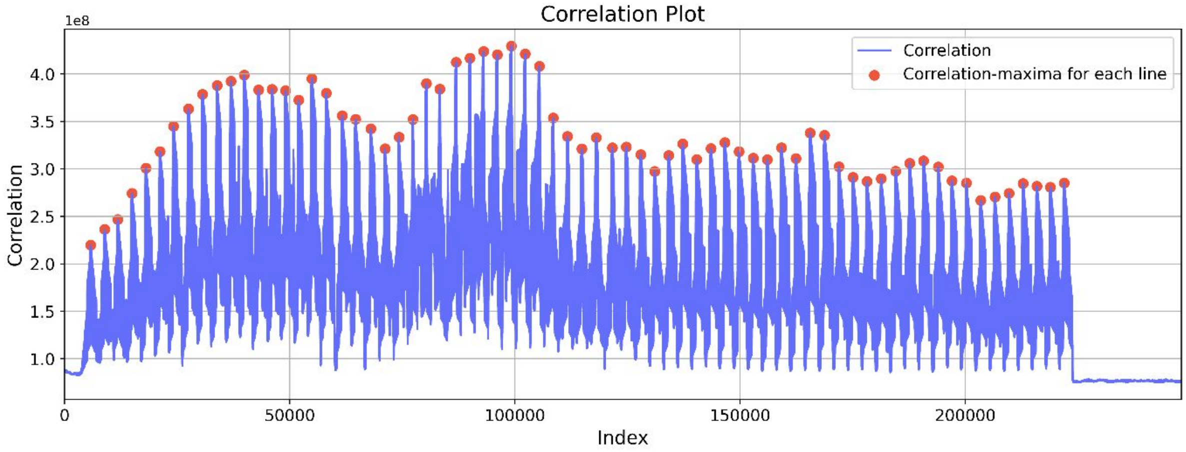

Figure S1. Correlation of the template signal with the transient ICP-MS signal.

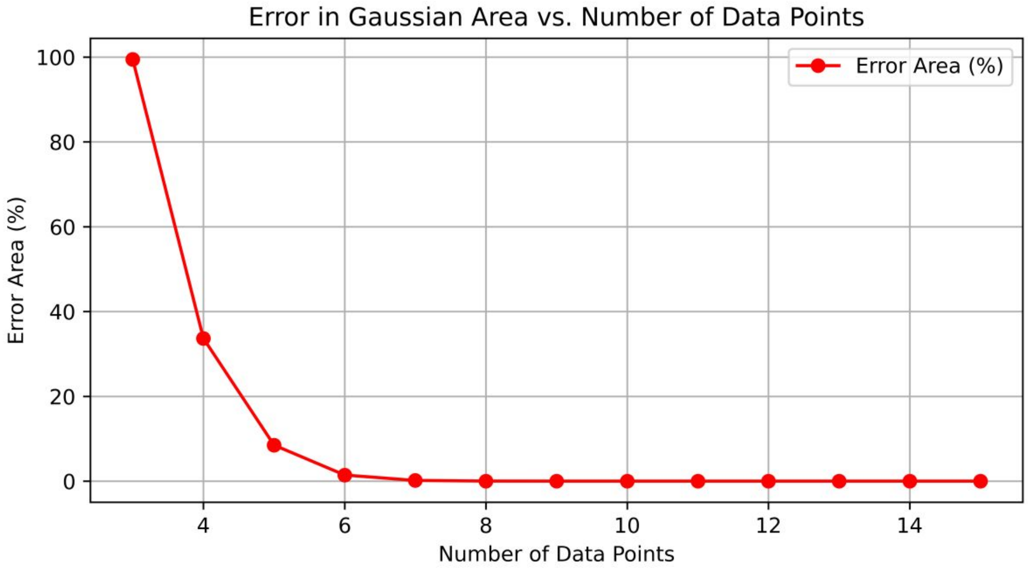

Figure S2. Relationship between the error of the integrated peak area (using the trapezoidal method) of a gaussian shaped signal depending and the number of recorded data points.

NIST SRM 612 measurements with „nano module“. The average peak integral and standard deviation from 35 peaks determined for ^{107}Ag using the “nano module” at dwell times of 0.025, 0.05, 0.075, 0.1, and 0.2 ms are shown in **Error! Reference source not found.**. It is observed that, as expected in this measurement mode, the average peak integral from 35 peaks of approximately 1900 a.u. is nearly constant for all investigated dwell times, showing only 1.8 % relative standard deviation between the different dwell times. The relative standard deviations within the 35 peaks measured for each specific dwell time, which is displayed as error bars in **Error! Reference source not found.**, shows values between 5 – 10 %. The highest value of 9.6 % is observed at the lowest dwell time (25 µs), which might be explained by the error derived from low counting statistics, since each 25 µs measurement window during a peak only counts an average of 11 ions with peak maximums varying between 25 and 40 ions per measurement window at that dwell time. Further, the average peak width at 1 % of the maximum varies between 7 and 8 ms corresponding to 280 – 320 data points, which is inversely proportional to the dwell time, while the average ion count per measurement window during the peak as well as the observed peak maxima increase directly proportional, leading to improved counting statistics with higher dwell times.

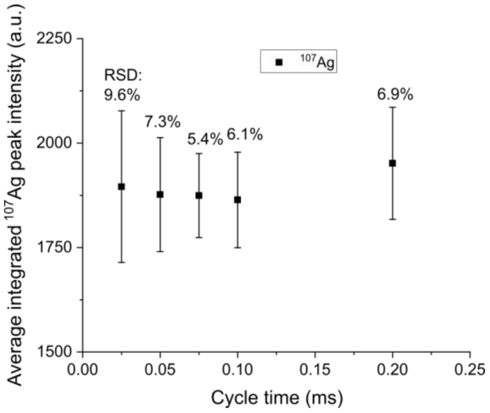

Figure S3. Average integrated ^{107}Ag peak intensity and RSDs measured at different dwell times using the “nano module” (single isotope acquisition without settling times) on NIST SRM 612.

Table S2. Relevant instrumental parameters and experiment characteristics.

**Experiment / Parameter**	NIST SRM 612 fundamental experiments	$SrTiO_3$ / $SrRuO_3$ test structure images	Bioimages of *Haematococcus pluvialis*
**Spot size***	20 x 20 µm²	2 x 2 µm²	5 x 5 µm²
**Repetition rate****	100 Hz	70 Hz	100 Hz
**Scan speed**	2000 µm/s	140 µm/s	500 µm/s
**Ablated area**	20 x 3000 µm² (0.06 mm²)	312 x 110 µm² (0.03432 mm²)	790 x 460 µm² (0.3634 mm²)
**Pixels**	1 x 150 (150)	156 x 55 (8580)	158 x 92 (14536)
**Measurement duration**	1.5 s	145 s	192 s

*corresponding to pixel size in imaging experiments

**corresponding to pixel acquisition rate in imaging experiments
